# A Novel Strategy of Ambiguity Correction for the Improved Faraday Rotation Estimator in Linearly Full-Polarimetric SAR Data

**DOI:** 10.3390/s18041158

**Published:** 2018-04-10

**Authors:** Jinhui Li, Yifei Ji, Yongsheng Zhang, Qilei Zhang, Haifeng Huang, Zhen Dong

**Affiliations:** College of Electronic Science and Engineering, National University of Defense Technology, Changsha 410073, China; jinhuinudt@gmail.com (J.L.); jyfnudt@gmail.com (Y.J.); zhangqilei@nudt.edu.cn (Q.Z.); h_haifeng@nudt.edu.cn (H.H.); dongzhen@nudt.edu.cn (Z.D.)

**Keywords:** synthetic aperture radar (SAR), Faraday rotation (FR), polarimetric SAR (PolSAR), FR angle (FRA) ambiguity, ambiguity correction

## Abstract

Spaceborne synthetic aperture radar (SAR) missions operating at low frequencies, such as L-band or P-band, are significantly influenced by the ionosphere. As one of the serious ionosphere effects, Faraday rotation (FR) is a remarkable distortion source for the polarimetric SAR (PolSAR) application. Various published FR estimators along with an improved one have been introduced to solve this issue, all of which are implemented by processing a set of PolSAR real data. The improved estimator exhibits optimal robustness based on performance analysis, especially in term of the system noise. However, all published estimators, including the improved estimator, suffer from a potential FR angle (FRA) ambiguity. A novel strategy of the ambiguity correction for those FR estimators is proposed and shown as a flow process, which is divided into pixel-level and image-level correction. The former is not yet recognized and thus is considered in particular. Finally, the validation experiments show a prominent performance of the proposed strategy.

## 1. Introduction

There is a growing trend in low-frequency synthetic aperture radar (SAR) systems for Earth observation, however, these systems are seriously affected by the ionosphere effects, such as group delay, phase advance, dispersion, scintillation, and Faraday rotation (FR) [1,2,3,4]. In particular, FR is generally known as a potential complication in the spaceborne polarimetric synthetic aperture radar (PolSAR) survey. At low frequencies, such as L-band and P-band, it becomes a more serious and non-negligible impact on polarimetric measurements [5,6,7,8,9,10]. As mentioned in published studies, FR angle (FRA) is inversely proportional to the square frequency and the maximal one-way FRA for L-band and P-band systems may reach as large as 40° and 321° [8,9,11], respectively. Therefore, it is necessary to evaluate, estimate and correct the FR effects on the spaceborne low-frequency PolSAR.

Authors have modeled and studied the FR effects on scattering matrix measurements by employing measured scattering matrix. A set of published FR estimators have been proposed to solve this issue of FR [8,9,11,12,13], and an improved FR estimator based on Bickel and Bates’s method has also been proposed, but the performance of the improved estimator has never been tested and proposed. In this paper, the improved estimator is tested in term of system noise, channel imbalance and cross-talk in detail [8,9,11]. However, all of published estimators suffer from a potential FRA ambiguity [5,8,9,11,12]. Some approaches have been devised to eliminate the FRA ambiguity, such as using specific terrain identities, phase unwrapping and FRA prediction [8,14]. Using specific terrain identities tends to be masked by system noise and phase unwrapping is generally lack of a baseline, but the FRA prediction, which can be renamed to image-level correction, is the most effective method for FRA ambiguity correction. However, pixel-level ambiguity of FRA estimates has not yet been recognized and studied, thereby this paper places much emphasis on it.

The paper aims to introduce an improved estimator and a novel strategy of FRA ambiguity correction for FR estimators, which is organized as follows. FR and a set of published FR estimators, including the improved estimator, are introduced in Section 2. Section 3 gives the performance analysis of the improved estimator in term of system noise, channel imbalance and cross-talk. A novel strategy to solve the FRA ambiguity is proposed in Section 4. The effectiveness of the correction strategy is verified by experiments based on real linearly full-polarimetric data processing in Section 5. Finally, we draw conclusions for the paper in Section 6.

## 2. Faraday Rotation Analysis

### 2.1. Faraday Rotation

A linearly-polarized electromagnetic wave propagating through the ionosphere splits into two circularly-polarized waves, which are equal in energy but rotate in opposite directions [7]. They recombine a new wave once leaving the ionosphere, but as a result, the new wave experiences a rotation of polarization plane compared with the original wave in the presence of the geomagnetic field and the free electrons in the ionosphere [7,8,9,12], and this phenomenon is well known as FR. The one-way FRA in radians can be approximately expressed as

(1) $$\Omega =K/f^{2}B\cos\theta \sec\varphi TEC$$

where K is a constant of value of $2.3648\times 10^{4}\left[A\cdot m^{2}/kg\right]$, f is the carrier frequency in hertz, B is the geomagnetic field intensity in webers per square meter, θ is the angle between the wave propagation and geomagnetic vector in radians, TEC is the local Total Electron Content (TEC) integrated along the propagation path, measured in TEC units (TECU) of $10^{16}$ electrons/$m^{2}$, and φ is the incident angle at the ionospheric altitude in radians.

Under the assumption that Ω has no obvious variation in the course of propagation, and ignoring the noise, the measured scattering matrix M can be written as [6,8,9,11,12]:

(2) $$\left[\begin{array}{cc} M_{hh} & M_{vh}\\ M_{hv} & M_{vv} \end{array}\right]=Ae^{j\varphi }\left[\begin{array}{cc} 1 & \delta_{1}\\ \delta_{2} & f_{1} \end{array}\right]\left[\begin{array}{cc} \cos\Omega & \sin\Omega \\ -\sin\Omega & \cos\Omega \end{array}\right]\left[\begin{array}{cc} S_{hh} & S_{vh}\\ S_{hv} & S_{vv} \end{array}\right]\left[\begin{array}{cc} \cos\Omega & \sin\Omega \\ -\sin\Omega & \cos\Omega \end{array}\right]\left[\begin{array}{cc} 1 & \delta_{3}\\ \delta_{4} & f_{2} \end{array}\right]$$

where A is the overall gain of the system; $e^{j\varphi }$ represents the comprehensive reflection consist of the phase delay of the propagation and the system-dependent phase; $\delta_{i},i=1,2,3,4,$ represents the channel cross-talk; $f_{i},i=1,2$ is the channel imbalance; $S_{hh},S_{hv},S_{vh},S_{vv}$ are the four elements of the true scattering matrix, $M_{hh},M_{hv},M_{vh},M_{vv}$ are the four elements of the measured scattering matrix, respectively.

Due to our focus on FR estimation, it is assumed that FR is the only measured error [6,7,9,10,12], and thus the measured scattering matrix can be written as:

(3) $$\left[\begin{array}{cc} M_{hh} & M_{vh}\\ M_{hv} & M_{vv} \end{array}\right]=\left[\begin{array}{cc} \cos\Omega & \sin\Omega \\ -\sin\Omega & \cos\Omega \end{array}\right]\left[\begin{array}{cc} S_{hh} & S_{vh}\\ S_{hv} & S_{vv} \end{array}\right]\left[\begin{array}{cc} \cos\Omega & \sin\Omega \\ -\sin\Omega & \cos\Omega \end{array}\right]$$

which can be further simplified as follows (assumed to be reciprocal, that is, $S_{hv}=S_{vh}$)

(4) $$\begin{array}{l} M_{1}=M_{hh}=S_{hh}\cos^{2}\Omega -S_{vv}\sin^{2}\Omega \\ M_{2}=M_{hv}=S_{hv}-(S_{hh}+S_{vv})\sin(2\Omega )/2\\ M_{3}=M_{vh}=S_{hv}+(S_{hh}+S_{vv})\sin(2\Omega )/2\\ M_{4}=M_{vv}=S_{vv}\cos^{2}\Omega -S_{hh}\sin^{2}\Omega \end{array}$$

Obviously, the measured scattering matrix doesn’t follow the backscatter reciprocity principle of $M_{hv}=M_{vh}$, unless Ω=0, which indicates the necessity of FR estimation.

### 2.2. Faraday Rotation Estimators

Under the assumption of the backscatter reciprocity and azimuthal reflection symmetry principles, several FR estimators have been proposed to solve this issue of FR. Derived from the covariance matrix of the circularly-polarized basis, the Bickel and Bates’ estimator [13], B&B for short, can be written as follows:

(5) $$\Omega_{B\&B}=\frac{1}{4}\arg(Z_{21}\times Z_{12}^{*})$$

where $Z_{21}$ and $Z_{12}$ are given by

(6) $$\left[\begin{array}{cc} Z_{11} & Z_{12}\\ Z_{21} & Z_{22} \end{array}\right]=\left[\begin{array}{cc} 1 & j\\ j & 1 \end{array}\right]\left[\begin{array}{cc} M_{hh} & M_{vh}\\ M_{hv} & M_{vv} \end{array}\right]\left[\begin{array}{cc} 1 & j\\ j & 1 \end{array}\right]$$

Freeman has also devised two FR estimators [11], but the first estimator is actually impractical for the argument of $\tan^{-1}$ may be complex since the existence of system noise. The second estimator, F2 for short, which is realized by adopting the spatial averaging method, and can be written as:

(7) ΩF2=±12tan−1〈(Mvh−Mhv)(Mvh−Mhv)*〉〈MhhMhh*〉+〈MvvMvv*〉+2Re(〈MhhMvv*〉)

where Re(⋅) obtains the real part of a complex argument, and 〈⋅〉 represents the spatial averaging method.

Based on the polarimetric covariance matrix (PCM) of the measured scattering matrix, Li Li has proposed two estimators [9], L1 and L2 for short, respectively. Experimental results show that the L1 estimator has complementary performances relative to the B&B estimator, and can be expressed as follows:

(8) ΩL1=12tan−1(Re(C13+C24−C12−C34)C11−C44)

where $C_{pq}=\langle M_{p}M_{q}^{*}\rangle$ is the covariance between the channel p and the channel q, and p and q take the values of 1, 2, 3, 4, respectively.

Jie Chen has also derived a set of FR estimators from PCM [8], and of which the third estimator, which is abbreviated as CHJ3, shows relatively better performances than other estimators and can be written as

(9) ΩCHJ3=12tan−1(Im(C13+C34−C12−C24)/2Im(C14))

where Im(⋅) obtains the imaginary part of a complex argument.

### 2.3. An Improved Estimator

As mentioned above, the spatial averaging method is adopted to reduce the influence of the system noise, and the spatial averaging method can also be applied to the B&B estimator, which can be rewritten as follows

(10) $$\Omega_{\langle B\&B\rangle }=\frac{1}{4}\arg\left(\langle Z_{21}\times Z_{12}^{*}\rangle \right)$$

combined with the PCM and (5), the argument of $\langle Z_{21}\times Z_{12}^{*}\rangle$, which is named as $Z_{power}$, and can be written as

(11) $$\begin{array}{cc} Z_{power} & =\langle Z_{21}\times Z_{12}^{*}\rangle =(C_{11}-C_{22}-C_{33}+C_{44}+C_{23}+C_{32}+C_{14}+C_{41})\\ & +j\times (C_{13}+C_{31}+C_{34}+C_{43}-C_{12}-C_{21}-C_{24}-C_{42}) \end{array}$$

and the corresponding estimator can be written as

(12) $$\begin{array}{cc} \Omega_{improve} & =\frac{1}{4}\arg(Z_{power})\\ & =\frac{1}{4}\arg\left(\begin{array}{l} \left(C_{11}-C_{22}-C_{33}+C_{44}+C_{23}+C_{32}+C_{14}+C_{41}\right)\\ +j\times (C_{13}+C_{31}+C_{34}+C_{43}-C_{12}-C_{21}-C_{24}-C_{42}) \end{array}\right) \end{array}$$

### 2.4. FR Estimation Based on Real Data

The improved estimator is verified by four sets of ALOS PALSAR data, which are obtained from Bohai of China in December 2007, Western Mongolia in June 2007, South Tibet of China in March, 2007, and Alaska in June 2007, respectively. The average values of FRA estimates are listed in Table 1, it can be seen from Table 1 that the average values of FRA estimates of the improved estimator are close to these of other estimators, which means an effective estimator. In addition, the average values of FRA estimates of the improved estimator are closer to these of the B&B estimator, which is usually considered as the most robust estimator.

Besides, it can also be seen that the overestimation occurs in the F2 estimator especially when the true FRA near 0°, which is consistent with the published results [9], and may be caused by the system noise.

## 3. Performance Analysis

### 3.1. Experimental Parameters

**Figure 1:**
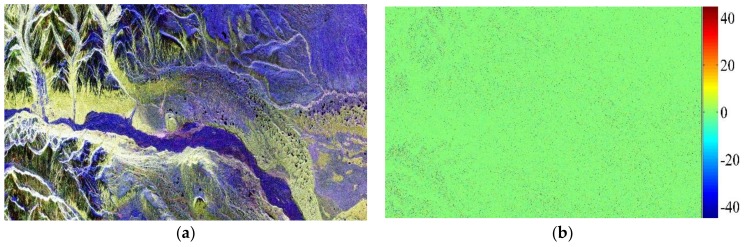
(**a**) ALOS PALSAR polarimetric image of a scene in Alaska (Pauli decomposition); (**b**) FRA Estimates from Alaska data.

A real full-polarimetric ALOS PALSAR data obtained over Alaska on 16 October 2006, whose local FRA is about 0°, is selected as the base data to perform the following experiments. The scene data is composed of mountains and forests, and can be shown as Figure 1a by Pauli decomposition. Figure 1b illustrates the FRA estimates of the Alaska data, and compared with the Figure 1a, it can be seen that the improved estimator has certain degree of dependence on the scattering characteristics of the target [9].

In addition, spatial averaging method is applied to the F2 estimator by a box car filter of 10×10 pixels [14], and for other estimators, the FRA estimates are also smoothed by a box car filter of the same size for fair comparison.

Various influencing factors, including system noise, channel imbalance and cross-talk, are injected into the real data according to (2) in turn. Combined with the actual situation, the signal to noise ratio (*SNR*) is defined as

(13) $$SNR=\left(\langle S_{hh}S_{hh}^{*}\rangle +2\langle S_{hv}S_{hv}^{*}\rangle +\langle S_{vv}S_{vv}^{*}\rangle \right)/\left(\langle N_{hh}N_{hh}^{*}\rangle +2\langle N_{hv}N_{hv}^{*}\rangle +\langle N_{vv}N_{vv}^{*}\rangle \right)$$

and for convenience, the noise power of all channels is set to equal, which means

(14) $$N_{hh}=N_{hv}=N_{vh}=N_{vv}$$

in addition, some reasonable assumptions are also made, in concrete, the SNR is varied from 0 to 20 dB; $f_{1}=f_{2}=f$, and the amplitude of f is varied from 0 to 1 dB, while the phase of f is varied from 0° to 10°; $\delta_{1}=\delta_{2}=\delta_{3}=\delta_{4}=\delta$, and the δ is varied from −40 to −10 dB.

Experiments are divided into two groups. In the first group, the effects of influencing factors on FR estimation for a given true FRA are studied, and the true FRA is set to 10° in this paper; in the second group, the effects of true FRA on FR estimation for the given influencing factors are studied. Besides, the performance of estimators is verified by the estimation biases between the average values of FRA estimates (for short $\bar{FRA}$) and the true FRA (for short tFRA), which can be defined as

(15) $$bias=\bar{FRA}-tFRA$$

### 3.2. Effects of System Noise

**Figure 2:**
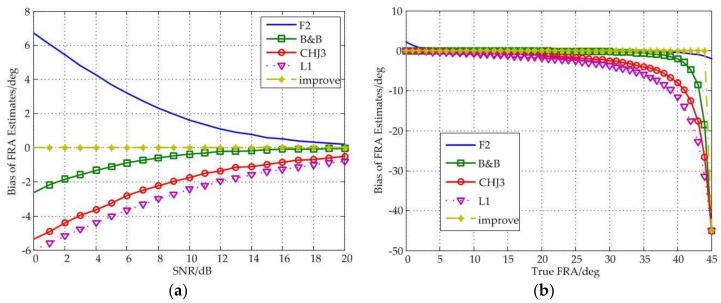
(**a**) Bias of FRA estimates as a function of SNR under different estimators; (**b**) Bias of FRA estimates as a function of true FRA under different estimators.

Figure 2 shows that the improved estimator has strong robustness. Experimental results indicate that the performance of the improved estimator is generally not affected by system noise, and when true FRA is less than 44°, the improved estimator maintains the bias to 0°.

It should also note that the estimation accuracy of the F2 estimator at 0° is poor, which is very likely to be caused by system noise. In addition, when the value of SNR is more than 3 dB and the true FRA is less than 30°, the biases of all estimators are no more than 5°, which is acceptable for most PolSAR applications [15]. Besides, all estimators fail near 45°.

Overall, in the influence of system noise, the improved estimator has the best performance.

### 3.3. Effects of Channel Imbalance

**Figure 3:**
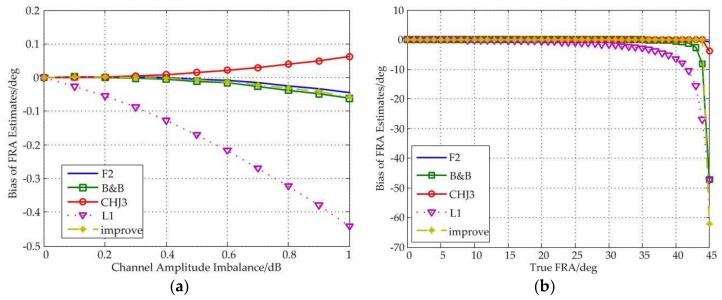
(**a**) Bias of FRA estimates as a function of channel amplitude imbalance under different estimators; (**b**) Bias of FRA estimates as a function of true FRA under different estimators.

Figure 3 shows the relationship between the estimation performance and the amplitude imbalance. It can be seen from the figure that the F2 estimator and the CHJ3 estimator have better performance. The improved estimator still shows great robustness. On the one hand, no matter the value of amplitude imbalance is, the bias of the improved estimator is limited to less than 0.1°; on the other hand, the performance of the improved estimator is basically not affected by the true FRA.

In addition, all of these estimators are basically unaffected by the amplitude imbalance, and even the amplitude imbalance is 1 dB, the biases of all estimators are no more than 0.5°; when the true FRA is less than 35°, the biases are no more than 5°.

Besides, all estimators fail near 45°.

**Figure 4:**
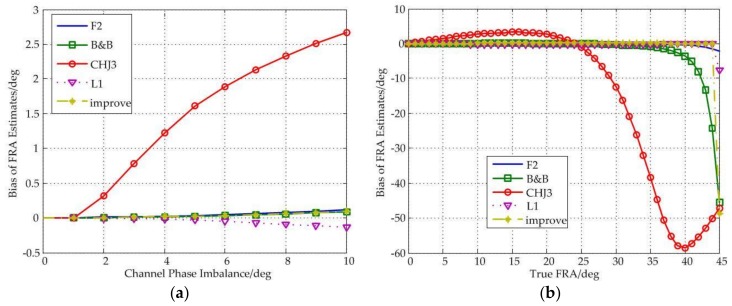
(**a**) Bias of FRA estimates as a function of channel phase imbalance under different estimators; (**b**) Bias of FRA estimates as a function of true FRA under different estimators.

In Figure 4, the influence of phase imbalance on FR estimation is analyzed. A conclusion can be derived from the figure is that the improved estimator, the F2 estimator and the L1 estimator have better and substantially the same performance, and no matter how the phase imbalance changes, the bias is always close to 0°, and when the true FRA is less than 44°, the biases are also around 0°.

In addition, we can also find that the CHJ3 estimator is very sensitive to phase imbalance and true FRA, and especially when the phase imbalance is 10°, the bias can be up to 2.7°, and when the true FRA is more than 27°, the bias even exceeds 5°, which will seriously affect the imaging performance and quality.

Besides, all estimators fail near 45°.

### 3.4. Effects of Cross-Talk

**Figure 5:**
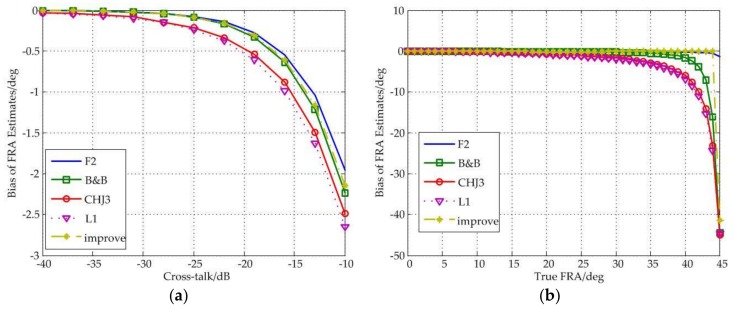
(**a**) Bias of FRA estimates as a function of cross-talk under different estimators; (**b**) Bias of FRA estimates as a function of true FRA under different estimators.

The influences of channel cross-talk on FR estimation can be drawn from Figure 5. Obviously, all kinds of estimators suffer from the cross-talk, but the new estimator also shows better performance, and is able to maintain a small bias regardless of the increasing values of the cross-talk, for example, when the values of cross-talk are no more than −10 dB, the bias of the improved estimator are less than 2.5°; when the true FRA is less than 44°, the bias is close to 0°.

In addition, it should also be noted that the F2 estimator has the best performance in term of cross-talk, under the all values of the cross-talk and true FRA, the F2 estimator has the smallest bias among these estimators.

Besides, all estimators fail near 45°.

It can be seen from the above experimental results that the improved estimator shows the best performance in the aspect of the system noise; the F2 estimator shows the best performance in term of the amplitude imbalance and the cross-talk; the improved estimator, the F2 estimator and the L1 estimator all show better performance in term of the phase imbalance. In fact, influencing factors, except system noise, can be calibrated to a negligible level, and it can be concluded that the improved estimator has the best performance.

## 4. FRA Ambiguity

### 4.1. FRA Ambiguity Analysis

As mentioned above, all estimators fail near 45°, which means all the published estimators suffer from the problem of FRA ambiguity. For example, the improved estimator has the FRA ambiguity of kπ/2, which means whatever the true FRA is, the improved estimator imposes the range of ±45° on FRA estimates, where k is an unknown integer.

The improved estimator is selected as the experimental object for its most robust performance compared with other estimators, and the real data obtained over Alaska is also used in the following experiments. In addition, averaging by a box car filter is a practical approach to depress system noise and remain possible FRA variations within image, which is necessary for the following correction experiments of the FRA ambiguity, and a box car filter of 10×10 pixels is also selected [14].

**Figure 6:**
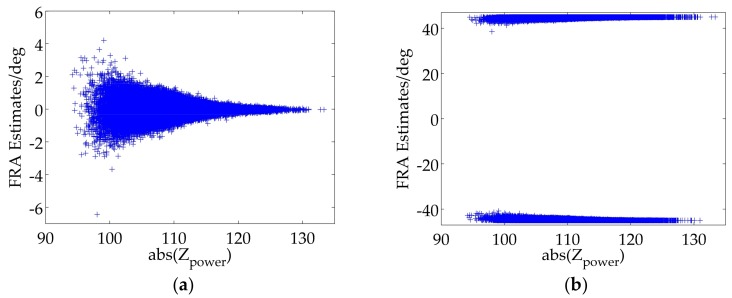
(**a**) FRA estimates for the original real data; (**b**) FRA estimates as the true FRA is set to 45°.

FRA estimates can be illustrated as scatter distribution diagrams versus $abs(Z_{power})$ [14], which can be considered to be the signal to noise ratio (SNR). The FRA estimates of all blocks are illustrated in Figure 6a. It is obviously shown that those blocks with higher SNR have more accurate FRA estimates closer to the average value. It also shows that the average value of FRA estimates of the original real data is approximate to 0°. Thereby, we inject a true FRA, which is set to 45°, into the original real data by using (3), and the corresponding experimental result is shown in Figure 6b. Unluckily, an unexpected distribution of FRA estimates occurs and the average value of FRA estimates is about 0.9256°, which implies an unsuccessful FR estimation and further proves the existence of FRA ambiguity.

### 4.2. Pixel-Level and Image-Level Ambiguity

Based on (1), the largest value of one-way FRA can be up to 40° at L-band and 321° at P-band, respectively, which means there is no FRA ambiguity for L-band in theory. Nevertheless, stemming from the existence of system noise, FRA estimates of some image pixels or blocks are likely to exceed the range of ±45° especially when the true FRA is close to the boundary value, and thus folding to the another side of FRA estimates range as shown in Figure 6b. An estimation error occurs if directly averaging all FRA estimates, and this phenomenon can be called pixel-level ambiguity. Furthermore, the true FRA in P-band SAR data is likely to exceed the range, which may lead to a more intricate situation with the potential pixel-level ambiguity and a holistic ambiguity, which can be named as image-level ambiguity. To sum up, the FRA ambiguity correction should be divided into pixel-level ambiguity correction and image-level ambiguity correction, both of which are under our focus and should be further specified.

### 4.3. A Novel Strategy for FRA Ambiguity Correction

**Figure 7:**
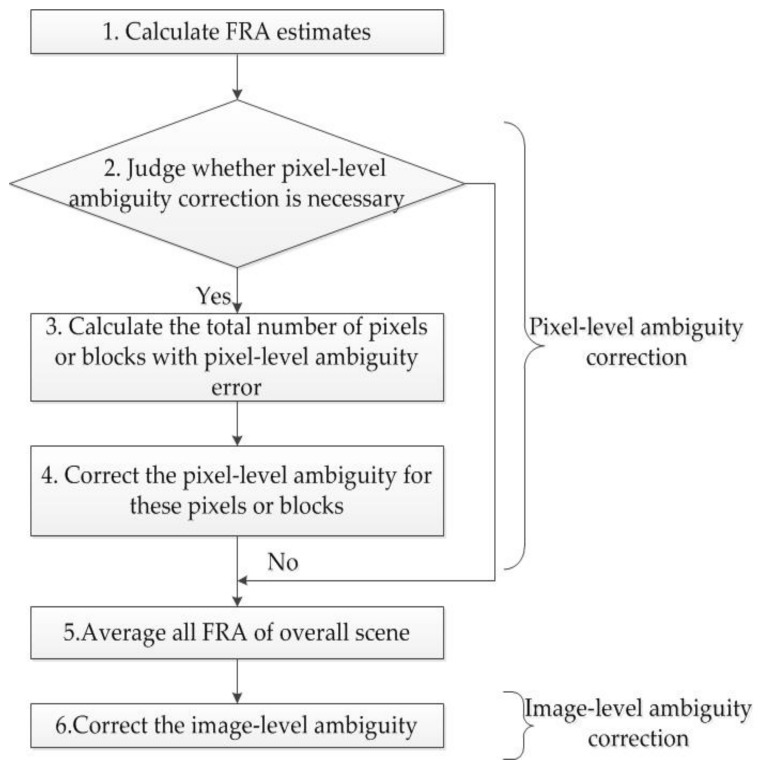
A flow chart of FRA ambiguity correction.

A procedure of FRA ambiguity correction is proposed to solve this issue, the flow chart of which is shown in Figure 7 and the steps are as follows.

Step I: FRA estimates are supposed to be firstly calculated via Equation (12). It is necessary for the next steps to use a box car filter of 10×10 pixels to get more centralized distributions.

Step II: It should be judged whether pixel-level ambiguity correction is necessary, which can be distinguished artificially or automatically. For example, pixel-level ambiguity correction is not required for Figure 6a, thus skipping to the Step V, but necessary for Figure 6b.

Step III: As is shown in Figure 6b, all FRA estimates are divided into one part closer to 45° and the other part closer to −45°. Total numbers of FRA estimates in the two parts are counted and denoted as N1 and N2, respectively.

Step IV: Based on the maximal likelihood estimation, the most likely event is expected, and the other should be revised. For example, if N1 is larger than N2, FRA estimates closer to −45° are considered to have the pixel-level ambiguity and should be added with 90°; otherwise, FRA estimates closer to 45° have the pixel-level ambiguity and should be subtracted by 90°.

Step V: The FRA is calculated by averaging all FRA estimates.

Step VI: According to the local time, height, longitude and latitude, the geomagnetic field can be obtained by employing International Geomagnetic Reference Field (IGRF), and vertical TEC values are derived from global ionosphere maps (GIMs). Therefore, based on the specific geometry of SAR system, FRA can be calculated as a crude prediction via Equation (1). Anyway, FRA prediction is a practical method to correct the image-level ambiguity.

## 5. Experimental Verification

**Figure 8:**
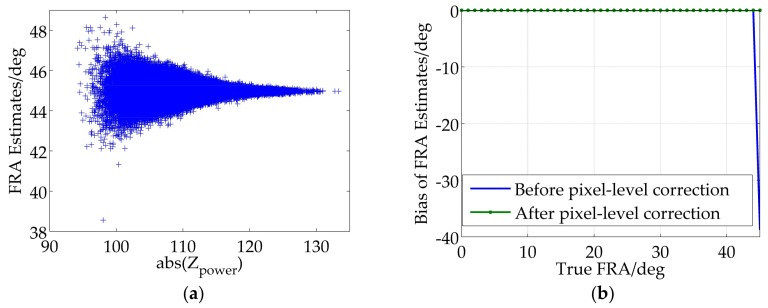
(**a**) FRA estimates after pixel-level correction as the true FRA is set to 45°; (**b**) Bias of FRA estimates as a function of true FRA after pixel-level correction.

Scatter distribution diagram of Figure 6b is corrected for the pixel-level ambiguity and illustrated in Figure 8a. It can be seen that FRA estimates are roughly focus on 45°, which indicates an accurate correction. Furthermore, in order to further verify the effectiveness of the pixel-level ambiguity correction, true FRA are set ranging 0° to 45°, which are tested by the full-polarimetric SAR real data, and estimation biases as a function of the true FRA are shown in Figure 8b. It can be seen that there are potential estimation biases for FR estimation due to the unsolved pixel-level ambiguity when the true value is close to 45°, and by applying the pixel-level ambiguity correction, the performance of the FR estimation is improved.

**Figure 9:**
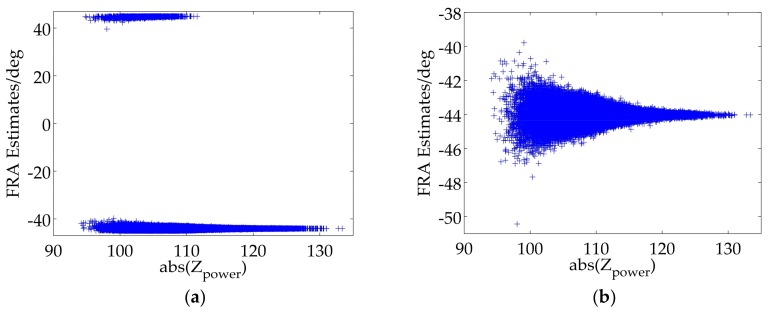
(**a**) FRA estimates as the true FRA is set to 46°; (**b**) FRA estimates after pixel-level correction as the true FRA is set to 46°.

Nevertheless, the true FRA is not merely limited within ±45° for the SAR systems operating at lower frequencies, such as P-band. A representative example is given in Figure 9, where the true value of FRA is set to 46° but estimated to be −44° by employing the pixel-level ambiguity correction. Furthermore, different true FRA larger than 45° are injected into the real data and FRA estimates are operated and results are given in Table 2. As shown in Figure 9 and Table 2, an estimation error of multiple 90° still remains after pixel-level ambiguity correction, which means the image-level ambiguity is still remained and needs to be further corrected.

Fortunately, a reference method can be applied to further correct the image-level ambiguity [8], which is described as follows

(16) $$\Omega_{i}=\Omega_{e}+round\left(\frac{\Omega_{p}-\Omega_{e}}{\pi /2}\right)\cdot \frac{\pi }{2}$$

where round(⋅) denotes rounding to the nearest integer; $\Omega_{i}$ means the FRA estimates by using image-level ambiguity correction in radians; $\Omega_{e}$ indicates the FRA estimates with the pixel-level ambiguity correction in radians; $\Omega_{p}$ is the FRA prediction in radians. As is above-mentioned in the Step VI, the FRA prediction is based on exterior database, such as GIMs and IGRF [14,16,17]. However, due to the lack of spaceborne P-band SAR real data, the image-level ambiguity correction cannot be verified, but it is theoretically feasible.

## 6. Conclusions

FR has a serious influence on the polarimetric application of spaceborne SAR systems especially operating at lower frequencies. FR estimation and correction for real data are necessary to improve polarimetric quality. An improved estimator is proposed and shows the strongest robustness. However, all proposed FR estimators including the improved estimator suffer from FRA ambiguity, and a novel strategy of FRA ambiguity correction based on the improved estimator is proposed to solve this issue, which can be divided into the pixel-level correction and the image-level correction. Experimental results based on the real data show the effectiveness of the pixel-level ambiguity correction. In addition, the idea of the FRA ambiguity correction can also be applied to other estimators.

There still are some issues to be solved. On the one hand, the effects of different scenes on the improved estimator should further be studied; on the other hand, verification of the image-level correction should also be realized as well as possible.

## Figures and Tables

**Table 1:** The average values of FRA estimates under all estimators.

Scene Area	Bohai of China	Western Mongolia	South Tibet	Alaska of American
F2	2.9253°	3.8961°	4.0675°	3.7893°
B&B	0.3183°	2.4603°	0.5129°	1.9149°
CHJ3	0.2816°	2.2970°	0.5767°	1.7350°
L1	0.2544°	2.2721°	0.4965°	1.6973°
improve	0.3099°	2.5461°	0.5904°	1.9300°

**Table 2:** FRA estimates for different true values larger than 45°.

True FRA	Folding FRA	Before Pixel-Level Correction	After Pixel-Level Correction
60°	−30°	−30.0022°	−30.0022°
95°	5°	4.9978°	4.9978°
135°	45°	0.9256°	44.9978°
136°	−44°	−43.6561°	−44.0022°
224°	44°	43.6095°	43.9978°
320°	−40°	−40.0022°	−40.0022°

## References

[1] Xu Z.W., Wu J., Wu Z.S. (2004). A survey of ionosphere effects on space-based radar. Waves Random Media.

[2] Li L., Zhang Y.S., Dong Z., Liang D.N. (2014). Ionospheric polarimetric dispersion effect on low-frequency spaceborne SAR imaging. IEEE Geosci. Remote Sens. Lett..

[3] Ji Y.F., Zhang Q.L., Zhang Y.S., Dong Z. (2017). L-band geosynchronous SAR imaging degradations imposed by ionospheric irregularities. China Sci. Inf. Sci..

[4] Ji Y.F., Zhang Q.L., Zhang Y.S., Dong Z. (2017). Analysis of background ionospheric effects on geosynchronous SAR imaging. Radioengineering.

[5] Qi R.Y., Jin Y.Q. (2007). Analysis of the effects of the Faraday rotation on spaceborne polarimetric SAR observations at P-band. IEEE Trans. Geosci. Remote Sens..

[6] Gail W.B. (1998). Effect of Faraday rotation on polarimetric SAR. IEEE Trans. Aerosp. Electron. Syst..

[7] Wright P.A., Quegan S. (2003). Faraday rotation effects on L-Band spaceborne SAR data. IEEE Trans. Geosci. Remote Sens..

[8] Chen J., Quegan S. (2010). Improved estimators of Faraday rotation in spaceborne polarimetric SAR data. IEEE Geosci. Remote Sens. Lett..

[9] Li L., Zhang Y.S. (2014). New Faraday rotation estimators based on polarimetric covariance matrix. IEEE Geosci. Remote Sens. Lett..

[10] Freeman A., Saatchi S.S. (2004). On the detection of Faraday rotation in linearly polarized L-band SAR backscatter signatures. IEEE Trans. Geosci. Remote Sens..

[11] Freeman A. (2004). Calibration of linearly polarized polarimetric SAR data subject to Faraday rotation. IEEE Trans. Geosci. Remote Sens..

[12] Wang C., Liu L. (2017). Improved TEC retrieval based on spaceborne PolSAR data. IEEE Trans. Geosci. Remote Sens..

[13] Bickel S.H., Bates R.H.T. (1965). Effects of magneto-ionic propagation on the polarization scattering matrix. Proc. IEEE.

[14] Meyer F.J., Nicoll J.B. (2008). Prediction, detection, and correction of Faraday rotation in full-polarimetric L-band SAR data. IEEE Trans. Geosci. Remote Sens..

[15] Rogers N.C., Quegan S. (2014). The Accuracy of Faraday Rotation Estimation in Satellite Synthetic Aperture Radar Images. IEEE Trans. Geosci. Remote Sens..

[16] Burgin M., Moghaddam M. Mitigation of Faraday Rotation effect for long-wavelength synthetic spaceborne radar data. Proceedings of the 2014 IEEE International Geoscience and Remote Sensing Symposium (IGARSS).

[17] Guo W., Chen J. Quantitative analysis of Faraday rotation impacts on image formation of spaceborne VHF/UHF-SAR. Proceedings of the 2014 IEEE International Geoscience and Remote Sensing Symposium (IGARSS).

